# Host-pathogen interaction in community-acquired pneumonia: a focus on the immune response

**DOI:** 10.3389/fcimb.2026.1731074

**Published:** 2026-02-11

**Authors:** Antonio Maria Ferriero, Raffaella Di Lella, Chiara Farroni, Alessandra Aiello, Antonino Giarratano, Matilde Todaro, Maria Grazia Bocci, Emanuele Nicastri, Delia Goletti

**Affiliations:** 1 Translational Research Unit, National Institute for Infectious Diseases Lazzaro Spallanzani-Istituto di Ricovero e Cura a Carattere Scientifico (IRCCS), Rome, Italy; 2 Department of Precision Medicine in Medical, Surgical and Critical Care (Me.Pre.C.C.), University of Palermo, Palermo, Italy; 3 Department of Anesthesia, Analgesia, Intensive Care and Emergency, University Hospital Policlinico Paolo Giaccone, Palermo, Italy; 4 Department of Health Promotion Sciences, Internal Medicine and Medical Specialties (PROMISE), University of Palermo, Palermo, Italy; 5 Azienda Ospedaliera Universitaria Policlinico “Paolo Giaccone” (AOUP), Palermo, Italy; 6 Anesthesia and Intensive Care Unit, National Institute for Infectious Diseases Lazzaro Spallanzani-Istituto di Ricovero e Cura a Carattere Scientifico (IRCCS), Rome, Italy; 7 Clinical Division of Infectious Diseases, National Institute for Infectious Diseases Lazzaro Spallanzani-Istituto di Ricovero e Cura a Carattere Scientifico (IRCCS), Rome, Italy

**Keywords:** adaptive immunity, bacterial pneumonia, CAP, community-acquired pneumonia, innate immunity, viral pneumonia

## Abstract

Community-acquired pneumonia (CAP) remains one of the leading causes of morbidity and mortality worldwide, affecting individuals of all ages. Various pathogens can cause this condition, and growing antibiotic resistance makes treatment more difficult while raising the risk of severe outcomes. Despite substantial advances in diagnostics, antimicrobial therapy, and supportive care, CAP continues to represent a significant clinical and public health challenge. In this review, we provide a comprehensive overview of CAP, summarizing key aspects of its epidemiology, pathogen frequency, and recent progress in diagnostic tools and biomarkers. We also describe the innate and adaptive immune responses involved in CAP, with a particular focus on pneumonia caused by *Staphylococcus aureus*, *Streptococcus pneumoniae*, *Haemophilus influenzae*, respiratory syncytial virus, severe acute respiratory syndrome coronavirus 2, and Influenza A and B viruses. A deeper understanding of CAP immunopathogenesis may support the development of improved diagnostic and therapeutic approaches for pneumonia management.

## 1 Introduction

**Figure 1:**
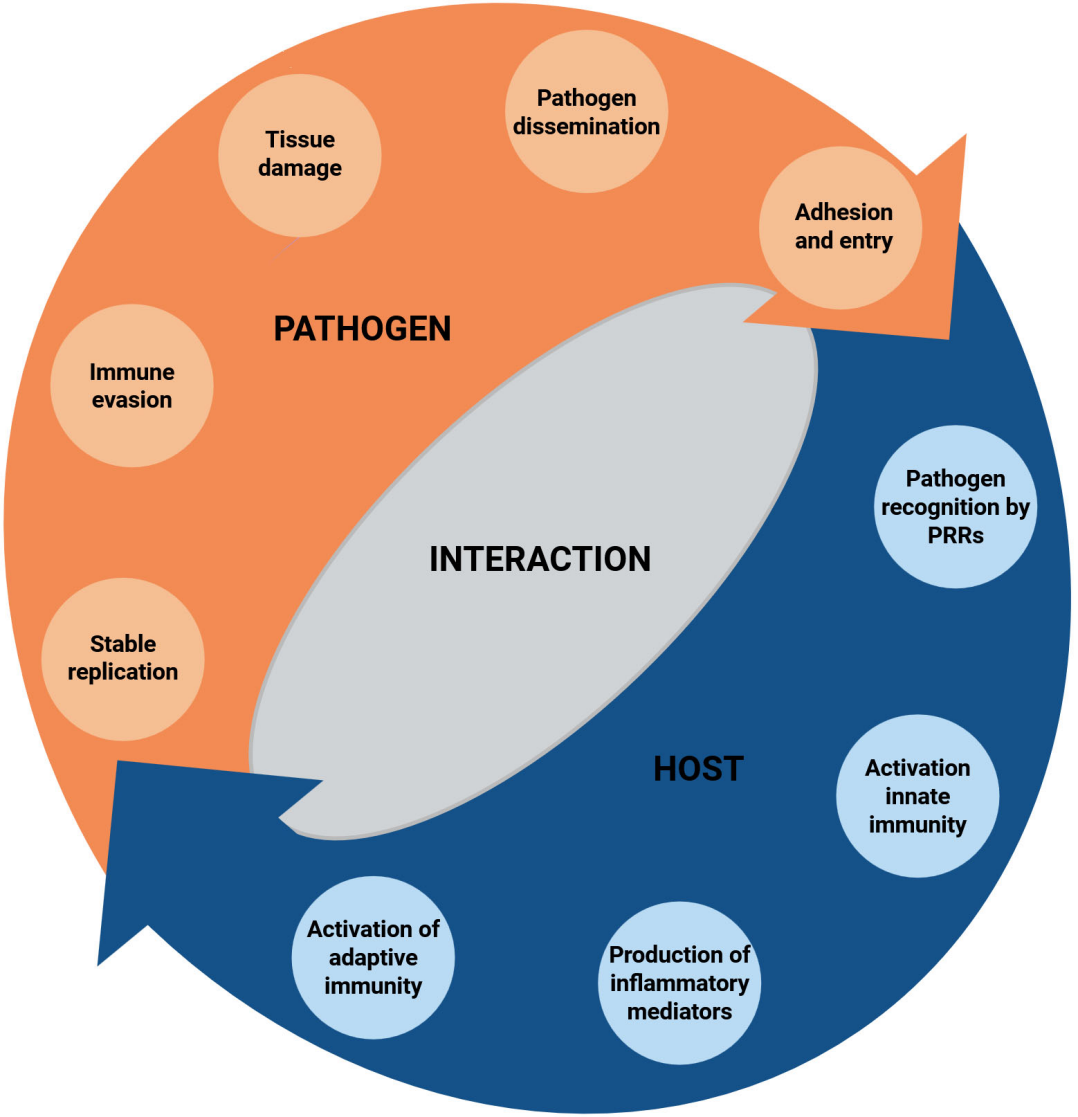
Dynamic interaction between host and pathogen. Key immune responses such as cell activation, cytokine immune factors release, and inflammation regulation are reported, along with the mechanisms by which pathogens can evade immunity, potentially leading to tissue damage and influencing disease progression. PRRs, pattern recognition receptors. Created with BioRender.com.

Community-acquired pneumonia (CAP) is a widespread and potentially serious respiratory infection that requires prompt and effective management to ensure optimal patient outcomes. CAP is defined as a sudden-onset infection affecting the alveolar regions of the lung parenchyma, occurring either in the community or within the first 48 hours of hospital admission (Le Bel et al., 2025). Although significant progress has been achieved in diagnostics, treatment, and patient care, CAP remains a major challenge for both clinical practice and public health. The first part of this review provides an overview of host-pathogen interactions in adult CAP (**Figure 1**), summarizing its epidemiology, current diagnostic approaches— with particular attention to emerging pathogen detection technologies—and the potential role of immune biomarkers in diagnosis and prognosis. The second part examines the innate and adaptive immunity to CAP, with a detailed discussion of host immunity to the bacterial (*Staphylococcus aureus, Streptococcus pneumoniae*, *Haemophilus influenzae*) and viral (respiratory syncytial virus, severe acute respiratory syndrome coronavirus 2, and Influenza A and B viruses) pathogens most frequently associated with this condition. Overall, understanding CAP pathogenesis, including the roles of inflammation, immune dysregulation, and long-term effects, is essential for advancing diagnostic tools, guiding personalized therapies, and ultimately reducing the global burden of this disease.

## 2 Epidemiology

Globally, the annual incidence of CAP varies significantly, ranging from 1.5 to 14 cases per 1,000 person-years, depending on geographic, seasonal, and demographic factors (Tsoumani et al., 2023; Vaughn et al., 2024). In Europe, during the decade 2011-2021, the incidence was estimated to range between 1.7 and 11.6 cases per 1,000 person-years, with over 1 million hospital admissions annually (Ostermann et al., 2014). In the United States, updated data from 2021 indicate that the incidence of CAP in adults is about 24.8 cases per 10,000 person-years, with a clear age-related increase (Vaughn et al., 2024). Among adults aged 65–79 years, the annual incidence reaches approximately 634 per 100,000, rising sharply to 16,430 per 100,000 in individuals aged 80 years or older (Vaughn et al., 2024). Hospitalization rates follow a similar trend: approximately 1,830 per 100,000 among those aged ≥65 years, compared to 199 per 100,000 in younger adults (Vaughn et al., 2024). Moreover, an estimated 10–20% of patients with CAP require hospitalization. Among these, in-hospital mortality poses a significant concern, varying between 5% and 20%, and reaching up to 50% in critically ill patients admitted to the intensive care unit (ICU) (Mandell, 2004; Vaughn et al., 2024). The 30-day mortality for hospitalized CAP patients averages 6%, but increases to 34% in cases of unresolved pneumonia or unfavorable initial clinical presentation (Peyrani et al., 2020). The diagnosis of CAP is challenging, as it relies on the integration of nonspecific clinical symptoms with radiological, microbiological, and biochemical findings. The coronavirus disease 19 (COVID-19) pandemic substantially impacted the CAP epidemiology, resulting in shifts in pathogen prevalence and a decrease in incidence attributed to non-pharmacological public health interventions. Concurrently, the situation accelerated advancements in diagnostic capabilities, especially in molecular testing, which has subsequently improved both the detection and surveillance of respiratory pathogens (Zanotto et al., 2025).

## 3 Common pathogens causing CAP

A broad spectrum of bacterial and viral pathogens can cause CAP, either as single agents or in combination. Among bacterial pathogens, in Western countries, the Gram-positive bacterium *Streptococcus pneumoniae (S. pneumoniae)* remains the predominant cause, identified in approximately 33–50% of confirmed CAP cases (Shoar and Musher, 2020; Markussen et al., 2024), followed by *Staphylococcus aureus (S. aureus)* in 4-10% of cases. Regarding Gram-negative bacteria, *Haemophilus influenzae (H. influenzae)* accounts for an estimated 7–16% of cases (Shoar and Musher, 2020), while *Enterobacteriaceae* are responsible for 4–10% of cases. Other Gram-negative bacteria, such as *Legionella pneumophila (L. pneumophila)*, *Pseudomonas aeruginosa (P. aeruginosa)*, and *Moraxella catarrhalis (M. catarrhalis)*, are less frequently isolated (<10% of cases) (Shoar and Musher, 2020). The prevalence of these pathogens varies by geographic region and season; for example, viral infections, particularly Influenza A and B, peak during the autumn and winter seasons, while in tropical or region-specific settings, bacterial pathogens such as *Klebsiella pneumoniae (K. pneumoniae)* or *Burkholderia pseudomallei (B. pseudomallei)* may be more common (Jain et al., 2015; Cilloniz et al., 2017). Respiratory viruses are detected in approximately 10–30% of immunocompetent adults hospitalized with CAP in Europe and the United States (Shoar and Musher, 2020). The most commonly isolated viruses include Influenza A and B (IAV and IBV), Rhinovirus, Respiratory Syncytial Virus (RSV), Human Metapneumovirus, and Parainfluenza virus. In approximately 25–35% of cases with a known etiology, these viruses may be involved in co-infections with bacterial pathogens (Holter et al., 2015; Escudero et al., 2022). Such co-infections are associated with worse clinical outcomes, including increased disease severity, prolonged hospitalization, and higher mortality rates, possibly due to complex interactions affecting host immune responses (McCullers, 2006; Barber et al., 2013). Additional emerging challenges include the increasing prevalence of multidrug-resistant pathogens, such as methicillin-resistant *S. aureus* (MRSA) and multidrug-resistant *P. aeruginosa*, underscoring the need for careful empirical antibiotic selection and ongoing epidemiological surveillance (Falcone et al., 2021).

## 4 Advances in diagnostics

The adoption of advanced molecular diagnostics, particularly multiplex PCR assays and next-generation sequencing (NGS) during the COVID-19 pandemic, has significantly improved the detection rate of respiratory viruses in CAP. Although a recent universal updated estimate of undetected pathogens is still lacking, recent studies indicate that the combined use of these technologies has significantly reduced non-detection rates, particularly in highly complex clinical settings (Liu et al., 2024), from approximately 60% (Torres et al., 2021) to 10% (Mitton et al., 2021). In recent years, the implementation of multiplex molecular syndromic testing—especially multiplex PCR panels—has significantly improved the diagnostic approach to respiratory infections, by enabling the simultaneous detection of multiple bacterial and viral pathogens, as well as antimicrobial resistance genes, from a single sample (e.g., sputum, tracheal aspirate, or bronchoalveolar lavage), with results available in a few hours. Currently, several multiplex panels are available for the rapid detection and characterization of specific pathogens in a clinical setting. These molecular diagnostic platforms can detect a broad spectrum of bacterial and viral agents from human samples, including *S. pneumoniae, H. influenzae, K. pneumoniae, Mycoplasma pneumoniae (M. pneumoniae), L. pneumophila*, influenza viruses, RSV, human metapneumovirus, and parainfluenza viruses (Buchan et al., 2020). Moreover, compared to the classical culture-based approach, these technologies show a detection rate of more than 90% (Falsey et al., 2024). In addition, it is also possible to identify some antimicrobial resistance genes [such as *mecA/C* (MRSA), KPC, NDM, OXA-48, and CTX-M (ESBL)] (Mitton et al., 2021). Other systems specifically designed for bronchoalveolar lavage specimens have a high sensitivity and specificity (more than 90%), and enable the simultaneous detection of multiple bacterial species, fungal pathogens (*Pneumocystis jirovecii*) (Klein et al., 2021), and resistance markers. Some multiplex assays for upper respiratory samples deliver results in about two hours and can detect a wide range of viral and bacterial targets. The sensitivity ranges from 85% to 95%, while specificity frequently exceeds 99%, supporting their utility in clinical practice. Some examples of commercial platforms are: the BioFire^®^ FilmArray Pneumonia Panel (PN and PNplus), the Unyvero LRT BAL Application, and GenMark ePlex Respiratory Pathogen Panel (Nijhuis et al., 2017; Babady et al., 2018).

## 5 Biomarkers in CAP

Currently, biomarkers specifically aimed at predicting or monitoring treatment failure in CAP remain limited. Preliminary evidence indicates that biomarkers such as C-reactive protein (CRP), procalcitonin (PCT), and various cytokines may be clinically useful. CRP is an acute-phase protein produced by the liver in response to inflammation, primarily mediated by Interleukin (IL)-6 (Gruys et al., 2005). Although CRP can contribute to supporting the diagnosis of pneumonia, it lacks specificity regarding the etiology (Le Bel et al., 2015). Values >100 mg/L are suggestive of bacterial infection, while values between 20–40 mg/L are more consistent with viral infections or non-infectious conditions (Le Bel et al., 2015). PCT is a precursor of calcitonin normally produced by thyroid C cells (Becker et al., 2010). During systemic bacterial infections, it is produced in large quantities throughout the body, especially by the liver, lungs, and leukocytes (Becker et al., 2010). PCT production is stimulated by pro-inflammatory cytokines [IL-1β, tumor necrosis factor-α (TNF-α), IL-6] and bacterial endotoxins, but inhibited during viral infections by interferon-γ (IFN-γ) (Becker et al., 2010). In addition to CRP and PCT, various cytokines are particularly relevant during pneumonia, including IL-1β, TNF-α, IL-6, IL-10, and transforming growth factor β (TGF-β) (Rendon et al., 2016). Notably, S100A8/A9 and CXCL8, primarily produced by specific inflammatory neutrophil subtypes, are central mediators of the cytokine storm during severe pneumonia (Xiao et al., 2025). Recently, some inflammatory biomarkers such as soluble triggering receptor expressed on myeloid cells-1 (sTREM-1), pro-adrenomedullin (proADM), and presepsin have been developed as relatively specific biomarkers for bacterial infection: *sTREM-1* measured in bronchoalveolar lavage fluid has shown excellent discrimination between patients with and without pneumonia: for example, in one NEJM study, sTREM-1 ≥5 pg/mL yielded sensitivity ~95-98% and specificity ~90% in detecting CAP (Gibot et al., 2004); *proADM* correlates strongly with established severity scores (e.g. PSI, CURB-65), predicts both short-term mortality and long-term outcomes, and adds prognostic value when used alongside clinical scores. In a meta‐analysis, elevated MR-proADM levels were associated with increased risk of complications/mortality in CAP (AUC ~0.74, RR ~6.2 for mortality) and improved discrimination when added to CRB-65/CURB-65 (Liu et al., 2016); presepsin (sCD14-ST) has been demonstrated to correlate with severity of CAP as defined by PSI and CURB-65, and to differentiate higher‐ vs lower‐risk patients: higher presepsin levels were found in non-survivors than survivors, and the biomarker yields moderate ROC AUC (≈0.70-0.73) in these settings (Ham and Song, 2019).

## 6 Key players in the immune response to CAP

**Figure 2:**
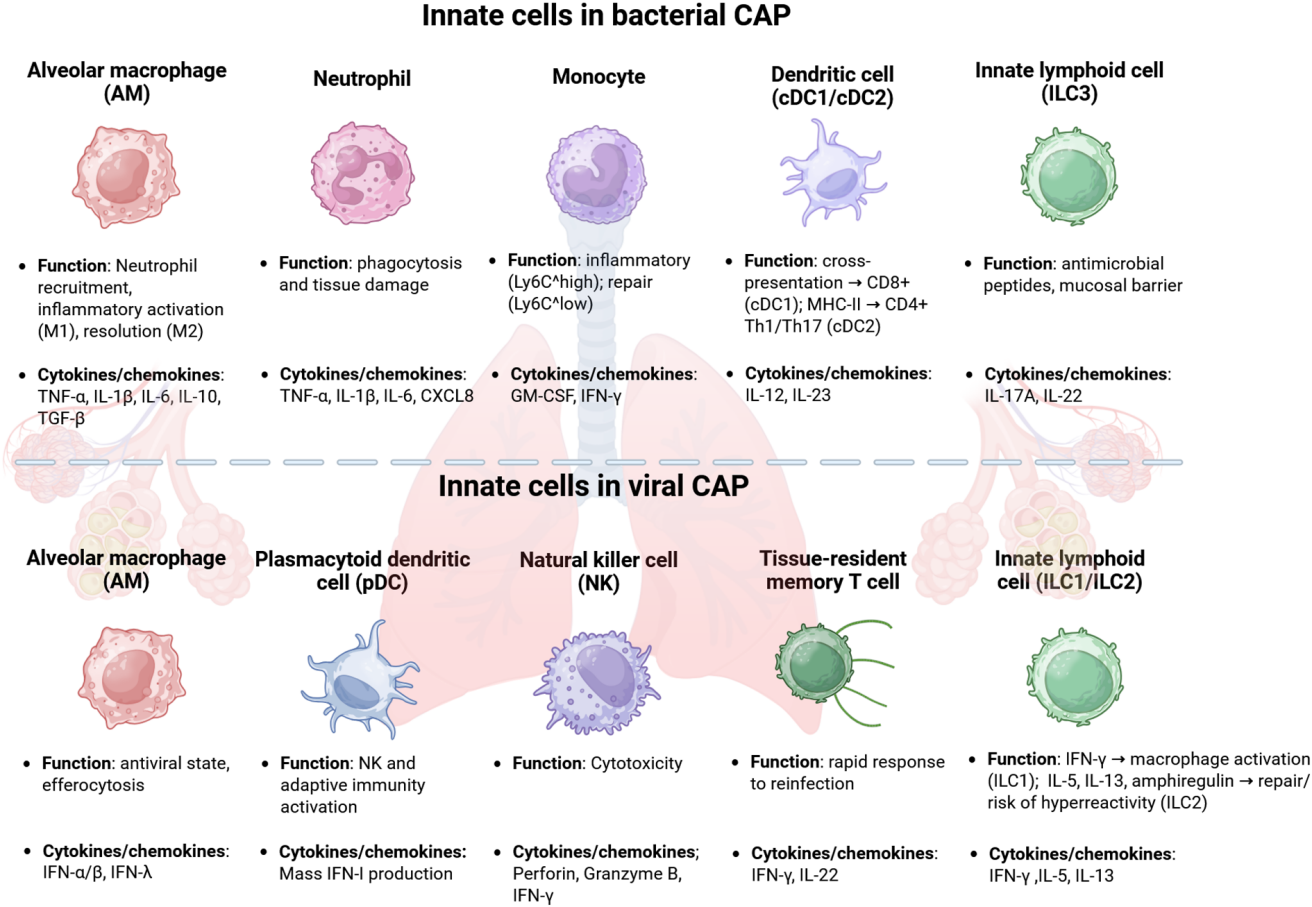
Innate immune cells involved in bacterial (up) and viral (down) lung infections. AM, alveolar macrophage; ILC, innate lymphoid cell; DC, dendritic cell; NK, natural killer; IL, interleukin; IFN, interferon; TNF, tumor necrosis factor; CXCL, C-X-C Motif Ligand; GM-CSF, Granulocyte-Macrophage Colony-Stimulating Factor. Created with BioRender.com.

**Figure 3:**
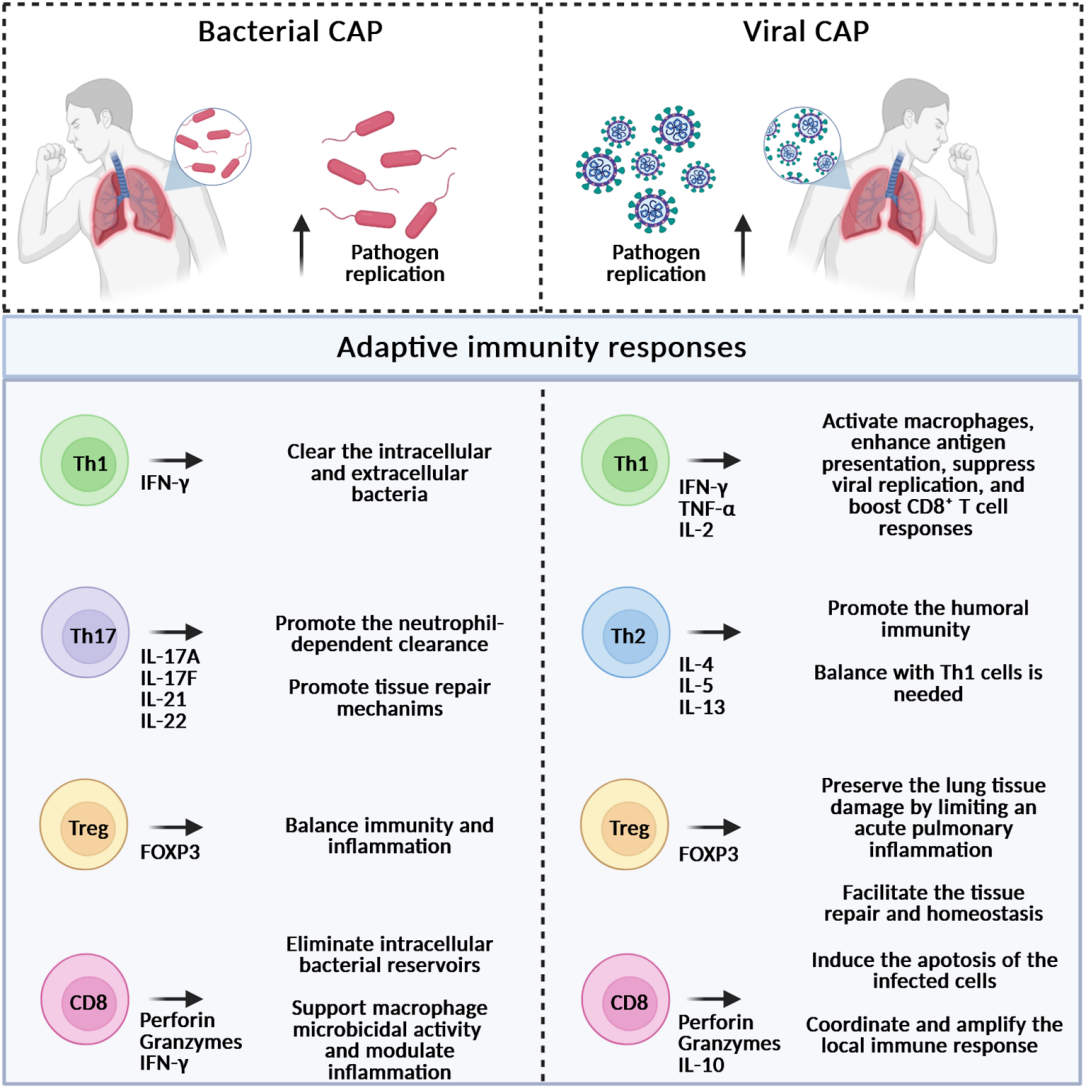
Adaptive immune responses during bacterial (left) and viral (right) lung infections. It is highlighted the role of the main lymphocyte subtypes involved. Th, T helper; Treg, T regulatory; CD, cluster of differentiation; IL, interleukin; IFN, interferon; TNF, tumor necrosis factor; FOXP3, forkhead boxP3. Created with BioRender.com.

Respiratory pathogens can spread between individuals through direct or indirect contact, droplets, or aerosols. Once in the nasopharynx, they can evade mucus clearance and adhere to the epithelial surface by employing various strategies (Torres et al., 2021). These include the expression of molecules that mimic host structures or undergo antigenic variation, allowing them to escape immune detection. After colonizing the nasopharynx, pathogens can reach the lower respiratory tract or, more rarely, enter the pleural space through hematogenous spread (Torres et al., 2021). Host immune status plays a significant role in determining the outcome of CAP, as evidenced by the increased severity observed in older individuals or in those with comorbidities such as HIV-coinfection and diabetes, or those receiving immunosuppressive therapies (Grifoni et al., 2023; Thong et al., 2025). The innate immune system represents the first line of defense against microbial invasion, providing a rapid but nonspecific response to a wide range of pathogens. Key cellular players involved in the innate immune response to CAP include monocytes/macrophages, neutrophils, dendritic cells (DCs), and natural killer (NK) cells, each contributing distinct and essential functions (**Figure 2**). Monocytes are circulating phagocytes that differentiate into macrophages or DCs upon recruitment to infected tissues (Kratofil et al., 2017). A particular subset of macrophages, alveolar macrophages (AMs), resides in the alveolar space, where they serve as primary sentinels against inhaled pathogens. AMs express multiple pattern recognition receptors (PRRs), including Toll-like receptors (TLRs), NOD-like receptors, and inflammasome components, enabling the detection of bacterial and viral products (Korkmaz and Traber, 2023). They can polarize toward a pro-inflammatory M1 phenotype or an anti-inflammatory M2 phenotype, thereby supporting pathogen clearance or inflammation resolution and tissue repair (Krausgruber et al., 2011; Kumar, 2020; Deng et al., 2023; Xu and Xie, 2023). Dysfunction or depletion of AMs is associated with severe disease and hyperinflammation. Neutrophils, other phagocytic cells, eliminate pathogens through phagocytosis, degranulation, reactive oxygen species (ROS) generation, and the formation of neutrophil extracellular traps (NETs) (Kumar, 2020). Although essential for bacterial clearance, excessive neutrophil activation contributes to tissue damage, pulmonary edema, and acute lung injury. Finally, NK cells contribute to the elimination of infected cells via cytotoxic mechanisms and IFN-γ secretion, supporting macrophage and DCs function (Cai et al., 2025). DCs act as professional antigen-presenting cells linking innate and adaptive immunity (De Leeuw and Hammad, 2024). The adaptive immune system consists of two arms: humoral and cell-mediated immunity (**Figure 3**). Humoral immunity is mediated by B lymphocytes, which produce antibodies to neutralize extracellular pathogens and participate in antigen presentation and immune regulation (Chen et al., 2022; Wang et al., 2022). B cell responses can be divided into canonical germinal center (GC) and noncanonical extrafollicular (EF) response (Piano Mortari et al., 2025). The EF response provides early protection by rapidly generating antibodies during the initial phase of infection. In contrast, the GC response establishes long-lasting humoral immunity through the production of memory B cells (MBCs), plasma cells (PCs), and high-affinity circulating antibodies (Piano Mortari et al., 2025). Cell-mediated immunity is orchestrated by T lymphocytes (Gopallawa et al., 2023). T cell responses are pivotal in resolving pneumonia by generating pathogen-specific effector functions (**Figure 3**). Following antigen recognition, T cells differentiate into two distinct subpopulations based on the expression of specific surface markers and their functional properties: CD4^+^ T cells, also known as helper T (Th) cells that include the subtypes Th1, Th2, Th17, T follicular helper (Tfh), and regulatory T cells (Tregs) (Smith et al., 2018; Qin et al., 2022; Gopallawa et al., 2023), and CD8^+^ T cells, also known as cytotoxic T lymphocytes (CTLs) (Chen et al., 2023). Briefly, Th1 cells produce IFN-γ, promoting macrophage activation and clearance of intracellular and extracellular pathogens (Chen and Kolls, 2013). Th2 cells support humoral immunity, but may impair bacterial clearance if excessively polarized (Contoli et al., 2015; Xiao et al., 2025). Th17 cells drive neutrophil recruitment and epithelial repair via the IL-17/IL-22 axis and are critical in both bacterial and viral pneumonia (Smith et al., 2018; Paiva et al., 2021). Tfh cells promote high-affinity antibody production and long-term humoral memory (Juno et al., 2020; Xiao et al., 2025). Tregs are a specialized subset of CD4^+^ T cells that play a crucial role in regulating tissue homeostasis by limiting acute pulmonary inflammation and promoting tissue repair and regeneration (Mangodt et al., 2015; Jovisic et al., 2023). They express the transcription factor FOXP3, which is essential for their development and suppressive function, and exert their regulatory activity through the secretion of anti-inflammatory cytokines, including IL-10 and TGF-β (Najafi-Fard et al., 2022; Carlini et al., 2023) as well as the expression of inhibitory receptors such as CD39, CTLA-4, and PD-1 (Jovisic et al., 2023; Thomas et al., 2023). CD8^+^ T cells contribute to viral clearance through cytotoxicity and cytokine production, while also limiting immunopathology via IL-10 secretion (Sun et al., 2009; Braciale et al., 2012; Schmidt and Varga, 2018; Mortezaee and Majidpoor, 2022). They eliminate infected cells during intracellular infections and support immune regulation and tissue repair during extracellular infections (Cho et al., 2000; Weber et al., 2011; Zhang et al., 2024). Unlike innate responses, adaptive immunity develops more gradually. Effective adaptive responses usually emerge about 6–10 days after the first antigen exposure (Sette and Crotty, 2021). Both CD4^+^ and CD8^+^ T cells expand and remain detectable for at least 6–8 months, reflecting the establishment of immunological memory (Petrone et al., 2023). Overall, host organisms use distinct recognition pathways and effector strategies to counteract viral and bacterial pathogens. Understanding these differences is fundamental for accurate diagnosis, targeted therapy, and the development of novel immunomodulatory approaches. The following sections will be focused on the immune response to *S. aureus*, *S. pneumoniae*, *H. influenzae*, RSV, severe acute respiratory syndrome coronavirus 2 (SARS-CoV-2), and IAV and IBV, the pathogens most often associated with CAP (**Tables 1**, **2**).

**Table 1:** Major innate immune mediators involved in CAP.

Pathogen	Primary recognition receptors (sensors)	Key innate effectors & cytokines/chemokines	Immune evasion mechanisms	References
*Staphylococcus aureus*	**TLR2/TLR1/TLR6:** Detect peptidoglycan/LTA; **NOD2:** Detects muramyl dipeptide.	**Neutrophils:** ROS (MPO) and NETosis; **Dendritic Cells**: Th17 priming **Cytokines/Chemokines:** CXCL8 (IL-8).	**CHIPS:** Blocks neutrophil chemotaxis; **SpA & SCIN**: Block opsonization; **LukAB:** Kills dendritic cells; **Catalase/Staphyloxanthin**: Neutralize ROS; **Nucleases:** Degrade NETs.	(Takeuchi et al., 1999; Brinkmann et al., 2004; Liu et al., 2005; Deshmukh et al., 2009; Zielinski et al., 2012; DuMont et al., 2013; Spaan et al., 2013; Scherr et al., 2014; Wilson, 2014; Nauseef, 2016)
*Streptococcus pneumoniae*	**TLR2**: Recognizes lipoteichoic acid; **TLR4**: Recognizes Pneumolysin (PLY); **NLRP3 Inflammasome**: Activated by pore formation.	**Neutrophils**: Essential for opsonophagocytosis; **Alveolar Macrophages**: Sentinel sensing; **Cytokines/Chemokines**: IL-1β, IL-18.	**Capsule**: Masks PAMPs; hinders phagocytic receptors; **PspC** (CbpA): Binds Factor H to inhibit complement; **Pneumolysin**: Induces ciliostasis.	(McNeela et al., 2010; Brown et al., 2002; Dockrell et al., 2003; Malley et al., 2003; Quin et al., 2007; Hyams et al., 2010)
*Haemophilus influenzae*	**TLR4**: Recognizes Lipooligosaccharide (LOS); **TLR2**: Detects lipoproteins.	**Epithelial Cells**: β-defensins, CXCL8; **Neutrophils**: Release NETs.	**Biofilm**: Mechanical shielding. **Sialylation**: Mimics the host to evade detection. **Protein E**: Recruits Factor H (complement inhibition).	(Hood et al., 1999; Shuto et al., 2001; Starner et al., 2006; Singh et al., 2010; Juneau et al., 2011; Van Eldere et al., 2014; Su et al., 2018)
Respiratory Syncytial Virus (RSV)	**TLR4/CD14:** Detects F protein; **TLR3/PKR & RIG-I:** Detect intracellular RNA.	**Neutrophils:** Excessive NETs causing obstruction. **Macrophages:** Shift to M2-like phenotype (persistence); **NLRP3:** IL-1β secretion.	**NS1:** Targets TRIM25 (inhibits RIG-I) & degrades STAT2; **SOCS:** Upregulated to block IFN signaling. **G Protein:** Mimics Fractalkine (CX3CL1) to block NK cells.	(Harrison et al., 1999; Kurt-Jones et al., 2000; Tripp et al., 2001; Groskreutz et al., 2006; Elliott et al., 2007; Moore et al., 2008; Shirey et al., 2010; Segovia et al., 2012; Cortjens et al., 2016; Ban et al., 2018)
SARS-CoV-2	**TLR7 & MDA5:** Recognize viral RNA.	**NLRP3:** Hyperactivation (cytokine storm); **Neutrophils:** Immunothrombosis via NETs; **NK Cells:** Exhaustion (NKG2A upregulation) **Cytokines/Chemokines**: Type I IFN;	**Nsp1:** Shuts down host mRNA translation; **ORF6:** Blocks STAT1/2 nuclear transport; **Macrophage Depletion:** Loss of resident AMs.	(Blanco-Melo et al., 2020; Carvelli et al., 2020; Liao et al., 2020; Mu et al., 2020; Schulte-Schrepping et al., 2020; Thoms et al., 2020; Zheng et al., 2020; Zuo et al., 2020; Aiello et al., 2021; Petrone et al., 2021; Petruccioli et al., 2021; Rodrigues et al., 2021; Aiello et al., 2022; Bastard et al., 2022)
Influenza Virus (IAV/IBV)	**RIG-I:** Detects 5'-ppp-RNA; **NLRP3:** Senses viral RNA; **NKp46:** Directly recognizes HA.	**NK Cells:** Direct lysis of infected cells; **Endothelial Cells:** Cytokine amplification; **Macrophages:** Rapid necrosis ("empty niche") **Cytokines/Chemokines**: Type I IFN.	**NS1 (IAV):** Inhibits TRIM25 and CPSF30; **NS1 (IBV):** Sequesters ISG15 and binds Nup98; **PB1-F2:** Exacerbates inflammation.	(Nemeroff et al., 1998; Mandelboim et al., 2001; Pichlmair et al., 2006; McAuley et al., 2007; Allen et al., 2009; Gack et al., 2009; Narasaraju et al., 2011; Teijaro et al., 2011; Ghoneim et al., 2013)

RSV, respiratory syncytial virus; SARS-CoV-2, severe acute respiratory syndrome coronavirus 2; IAV/IBV, influenza A/B virus; TLR, Toll-like receptor; NOD2, nucleotide-binding oligomerization domain-containing protein 2; NLRP3, NOD-, LRR- and pyrin domain-containing protein 3; CD, cluster of differentiation; RIG-I, retinoic acid–inducible gene I; MDA5, melanoma differentiation–associated protein 5; PKR, protein kinase R; ROS, reactive oxygen species; MPO, myeloperoxidase; NETs, neutrophil extracellular traps; AMs, alveolar macrophages; NK, natural killer; IL, interleukin; IFN, interferon; TNF, tumor necrosis factor alpha; CXCL8/IL-8, C-X-C motif chemokine ligand 8; LTA, lipoteichoic acid; LOS, lipooligosaccharide; PAMPs, pathogen-associated molecular patterns; HA, hemagglutinin; NA, neuraminidase; CHIPS, chemotaxis inhibitory protein of Staphylococcus aureus; SpA, staphylococcal protein A; SCIN, staphylococcal complement inhibitor; PLY, pneumolysin; PspC/CbpA, pneumococcal surface protein C/choline-binding protein A; NS1, non-structural protein 1; ORF6, open reading frame 6; PB1-F2, polymerase basic protein 1 frame 2; SOCS, suppressor of cytokine signaling; STAT, signal transducer and activator of transcription; ISG15, interferon-stimulated gene 15; NKG2A, natural killer group 2A receptor. The words of interest are highlighted in bold.

**Table 2:** Main adaptive immune responses to CAP pathogens: involved cell types, functions, and key cytokines.

Pathogen	Main cell type and function			Cytokines/chemokines	References
	CD4^+^ T cells	CD8^+^ T cells	B cells	Key cytokines	
*Staphylococcus Aureus*	**Th1-Th17:** macrophage activation, neutrophil recruitment and antimicrobial peptide production.	Eliminate infected host cells and restrict intracellular bacterial replication.	Antibody responses against surface-associated antigens and secrete toxins.	IFN-γ, IL-17	(Chen and Kolls, 2013; Armentrout et al., 2020; Mirzaei et al., 2020)
*Streptococcus Pneumoniae*	**Th1-Th17:** support macrophages, NK cells, neutrophils. **Th2:** promote B cell activation **Tregs:** maintain immune homeostasis.	Kill infected cells; Limit tissue damage; Contribute to lung repair	Implicated in neutralization and long-lasting humoral immunity.	IFN-γ; IL-4; IL-17	(Weber et al., 2011; Wilson et al., 2015; Brooks and Mias, 2018; Ramos-Sevillano et al., 2019; Zhang et al., 2024)
*Haemophilus Influenzae*	Reduce **Th1** responses in severe disease form; **Th2**-skewing is associated with impaired bacterial clearance.	**Tc1:** impaired. **Tc2:** predominates and are less effective at controlling infection.	Antigen-specific antibody responses, at the mucosal level; Secretory IgA: bacterial neutralization and opsonization.	IFN-γ/IL-4	(Rao et al., 1999; King et al., 2003; King and Sharma, 2015; Su et al., 2018)
Respiratory Syncytial Virus (RSV)	**Th1:** efficient viral clearance; **Th2** and **Th17:** contribute to RSV severity causing airway inflammation, mucus hypersecretion. **Tfh:** promote B cell activation.	Excessive responses contribute to tissue damage and delay viral clearance.	**PCs** differentiation; Mucosal IgA and Serum IgG targeting RSV G and F glycoproteins.	IFN-γ, IL-17, IL-4	(Larrañaga et al., 2009; Ruckwardt et al., 2009; Fulton et al., 2010; Lee et al., 2010; Mukherjee et al., 2011; Faber et al., 2012; Varga and Braciale, 2013; Christiaansen et al., 2014; Mangodt et al., 2015; Russell et al., 2017; Gambadauro et al., 2024; Georgakopoulou and Pitiriga, 2025)
SARS-CoV-2	**Tfh:** establish a robust humoral immune memory and aid CD8^+^ T cell responses.	Exhaustion phenotypes are associated with severe outcomes.	Dysregulated EF response in severe COVID-19 infection. **DNs B cells** and **short-lived PCs** expansion are linked to hyper-inflammation and tissue damage.	IFN-γ, IL-4, IL-17, IL-21	(Woodruff et al., 2020; Sette and Crotty, 2021; Turner et al., 2021; Lee et al., 2022; Silva et al., 2022; Aiello et al., 2023; Iwanaga et al., 2023; Petrone et al., 2023; Sette et al., 2023; Piano Mortari et al., 2025)
Influenza Virus (IAV/IBV)	**Th1**: enhancement of CTLs activity and macrophage responses. **Tfh:** B cell activation.	Perforin and granzymes (such as GrA) release; Apoptosis induction through death receptor–mediated pathways; Production of pro-immunoregulatory cytokines such as IL-10.	IAV-specific antibodies targeting HA and NA surface glycoprotein; **IgG:** predominant antibody class mediating systemic immunity and limiting disease severity **IgA:** crucial role at respiratory mucosal surfaces and reduces viral transmission.	IFN-γ, TNF-α IL-4, IL-17, IL-21, IL-10	(Braciale et al., 2012; Chen et al., 2018; Koh et al., 2023; Gambadauro et al., 2024)

RSV, respiratory syncytial virus; SARS-CoV-2, severe acute respiratory syndrome coronavirus 2; IAV/IBV, influenza A/B virus; CD, cluster of differentiation; Th, T helper; Tfh, T follicular helper; Tregs, regulatory T cells; Tc, cytotoxic T cells; CTLs, cytotoxic T lymphocytes; EF, extrafollicular; PCs, plasma cells; DN B cells, double-negative B cells; IFN, interferon; IL, interleukin; TNF, tumor necrosis factor alpha; Ig, immunoglobulin; HA, hemagglutinin; NA, neuraminidase; NK, natural killer; GrA, granzyme A. The words of interest are highlighted in bold.

### 6.1 Immune response to *Staphylococcus aureus*

Among Gram-positive bacteria, *S. aureus* represents an emerging cause of CAP (Zhang et al., 2018; Grousd et al., 2019). The management of S. *aureus*-associated CAP is increasingly challenging due to the evolving antibiotic resistance of circulating strains, including methicillin-resistant *S. aureus* (MRSA) and vancomycin-intermediate (VISA) and vancomycin-resistant (VRSA) strains (Bröker et al., 2014; Zhang et al., 2018). Clinically, *S. aureus*-associated CAP carries an important clinical burden, with up to 81% of affected patients requiring intensive care support (Grousd et al., 2019). The interaction between *S. aureus* and the innate immune system is a dynamic and complex interplay in which the outcome depends on the balance between rapid host recognition and the pathogen’s virulence and evasion factors. The immune response is initiated when resident sentinel cells, specifically keratinocytes, tissue-resident macrophages, and dendritic cells, detect pathogen-associated molecular patterns (PAMPs). TLR2 heterodimerizes with TLR1 or TLR6 to recognize staphylococcal cell wall components like peptidoglycan and lipoteichoic acid, while the cytosolic sensor NOD2 detects intracellular muramyl dipeptide (Takeuchi et al., 1999; Deshmukh et al., 2009). This recognition triggers downstream signaling via MyD88 and NF-κB, resulting in the secretion of pro-inflammatory cytokines and the chemokine CXCL8, which orchestrates the massive recruitment of neutrophils to the site of infection. Simultaneously, resident DCs play a pivotal role by internalizing the pathogen and linking innate and adaptive immunity. Upon activation, DCs migrate to draining lymph nodes to prime naïve T cells, specifically driving a Th17-polarized response, which is essential for mucosal defense against extracellular bacteria (Zielinski et al., 2012). While DCs organize the long-term defense, recruited neutrophils act as immediate effectors, extravasating via integrin activation and actin reorganization to phagocytose opsonized bacteria via IgG and C3b complement fragment. Following engulfment, assembly of the NADPH oxidase complex generates ROS, which are converted by myeloperoxidase (MPO) into hypochlorous acid, a potent microbicidal agent (Nauseef, 2016). Under high bacterial loads, neutrophils may also undergo NETosis, a programmed form of death in which neutrophils release DNA traps containing antimicrobial proteins to immobilize bacteria (Brinkmann et al., 2004). *S. aureus* has evolved multiple strategies to evade host immune defenses. To inhibit neutrophil recruitment, the bacterium secretes the Chemotaxis Inhibitory Protein of *S. aureus* (CHIPS), which blocks C5a and formyl peptide receptors. Additionally, Staphylococcal Protein A (SpA) and Staphylococcal Complement Inhibitor (SCIN) help mask the bacterium from phagocytic receptors and prevent opsonization by binding immunoglobulins and C3 convertases, respectively (Wilson, 2014). The pathogen also targets DCs to blunt the adaptive immune response; the bicomponent leukocidin LukAB has been shown to specifically bind to the CD11b integrin on human DCs, inducing lytic cell death and thereby terminating antigen presentation before it can effectively initiate (DuMont et al., 2013). Even when phagocytosed by surviving granulocytes, *S. aureus* can persist intracellularly by neutralizing ROS with catalase and the antioxidant pigment staphyloxanthin (Liu et al., 2005). It further evades immune defenses by secreting nucleases (Nuc) that degrade NETs and releasing pore-forming toxins like Panton-Valentine Leukocidin (PVL), which lyse neutrophils and induce tissue necrosis (Spaan et al., 2013). *S. aureus* persistence is further exacerbated by biofilm formation on medical devices or necrotic tissue, where the extracellular matrix and frustrated phagocytosis lead to a chronic inflammatory state dominated by collateral tissue damage rather than bacterial clearance (Scherr et al., 2014). As *S. aureus* infection progresses, the contribution of the adaptive immunity becomes increasingly prominent. Following antigen recognition, CD4^+^ T cells and CTLs coordinate bacterial clearance, support tissue repair, and contribute to long-lasting immunological memory (Chen and Kolls, 2013). Among CD4^+^ T cells, Th1 and Th17 subsets play critical roles in host defense against *S. aureus*: Th1 cells produce IFN-γ, enhancing macrophage activation and intracellular killing, whereas Th17 cells secrete IL-17, promoting neutrophil recruitment and antimicrobial peptide production at infection sites (Armentrout et al., 2020; Mirzaei et al., 2020). Although traditionally considered an extracellular pathogen, accumulating evidence indicates that *S. aureus* can persist intracellularly, particularly within macrophages. It can evade phagosomal killing, impair the phagolysosomal maturation, and translocate into the cytoplasm, thus behaving as a facultative intracellular pathogen. In this context, CD8^+^ T cells and NK cells are particularly relevant, as they can eliminate infected host cells and restrict intracellular bacterial replication (Armentrout et al., 2020). Humoral immunity also plays a significant role in controlling *S. aureus* infection (Mirzaei et al., 2020). Infection or colonization induces antibody responses against surface-associated antigens and secreted toxins, involving IgM, IgA, and IgG subclasses. However, *S. aureus* has developed multiple mechanisms to avoid antibody-mediated detection. SpA and the second immunoglobulin-binding protein (Sbi) bind the Fc region of antibodies, blocking opsonophagocytosis and interfering with complement activation (Armentrout et al., 2020; Mirzaei et al., 2020). Furthermore, SpA has been found to act as a B cell superantigen by binding the F(ab)2 region of the B cell receptor, inducing apoptosis, and thereby inhibiting effective antibody production (Armentrout et al., 2020).

### 6.2 Immune response to *Streptococcus pneumoniae*

*S. pneumoniae* is a Gram-positive bacterium that frequently colonizes the nasopharynx of both infants and adults (Cohen et al., 2011; Ramos-Sevillano et al., 2019; Gil et al., 2022). Over the past decades, the introduction of pneumococcal vaccines has significantly reduced the burden of pneumococcal infections; nevertheless, the pathogen remains responsible for approximately 10-15% of CAP cases (Cohen et al., 2011; Grousd et al., 2019; Gil et al., 2022). The incidence of pneumococcal pneumonia increases with advancing age, particularly in individuals over 65 years (Wilson et al., 2015). This increased susceptibility is mostly attributed to immunosenescence, which refers to the progressive decline in immune competence, which includes diminished CD4^+^ T cells proliferation and impaired antigen-specific responses, resulting in an inability to detect and eliminate pathogens (Smith et al., 2018). Furthermore, individuals with defects in adaptive immunity, whether due to genetic or acquired deficiencies in immunoglobulin production, or severe dysfunction following stem cell transplantation or HIV infection, demonstrate increased susceptibility to *S. pneumoniae* infections (Ramos-Sevillano et al., 2019). The innate immune response to *S. pneumoniae* serves as a crucial protective mechanism particularly within the lower respiratory tract. The colonization success or failure depends on the interplay between mucosal clearance processes and bacterial evasion strategies. The response is initiated when resident sentinel cells, AMs and airway epithelial cells detect the pathogen. Unlike the robust recognition of staphylococci, pneumococcal sensing is complicated by its polysaccharide capsule, which masks surface ligands. However, the detection of *S. pneumoniae* occurs via TLR2, which recognizes lipoteichoic acid, and uniquely through TLR4, which has been identified as the sensor for the cholesterol-dependent cytolysin pneumolysin (PLY) (Malley et al., 2003). Upon recognition, the pore-forming activity of PLY causes potassium efflux and cytosolic access for bacterial products, triggering NLRP3 inflammasome assembly in macrophages, and subsequent caspase-1-dependent processing and secretion of IL-1β and IL-18 (McNeela et al., 2010). These cytokines, together with pulmonary epithelial-derived signals, drive neutrophil recruitment into alveolar spaces, a hallmark of pneumococcal pneumonia. Effective clearance by recruited neutrophils and resident macrophages relies on opsonophagocytosis, which is facilitated by the deposition of C3b and CRP on the bacterial surface (Brown et al., 2002). However, *S. pneumoniae* has developed sophisticated strategies to circumvent host defenses and facilitate the development of invasive disease. The most significant virulence factor is the polysaccharide capsule, which acts as a physical shield to mask PAMPs from TLRs and sterically hinder interactions between phagocytic receptors (e.g., FcγR and CR3) and opsonins on the bacterial cell wall, thereby blocking non-opsonic phagocytosis (Hyams et al., 2010). Beyond passive shielding, pneumococcus actively manipulates complement; the pneumococcal surface protein C (PspC, also known as CbpA) binds the host negative regulator Factor H, recruiting it to the bacterial surface to degrade C3b and inhibit the alternative complement pathway (Quin et al., 2007). In addition, the pathogen interferes with the lung’s physical defenses; sub-lytic amounts of pneumolysin cause ciliostasis in the respiratory epithelium, stopping mucociliary clearance. At the same time, neuraminidase (NanA) breaks down mucus components, revealing epithelial receptors that facilitate adhesion (Hyams et al., 2010). Once internalized by macrophages, *S. pneumoniae* can induce apoptosis rather than effective killing, acting as a “Trojan horse” to deplete the sentinel cells and facilitate systemic dissemination (Dockrell et al., 2003). The adaptive immune response is essential for controlling *S. pneumoniae* infection, involving various CD4^+^ T cell subsets that support B cell antibody production and prompt tissue-resident memory T cell responses (Wilson et al., 2015). Following nasopharyngeal colonization, T cells respond to pneumococcal antigens mainly through Th1, Th2, and Th17 cell subsets (Brooks and Mias, 2018). Th1 cells produce IFN-γ, promoting the activation and recruitment of innate immune cells such as macrophages and NK cells; Th2 cells secrete IL-4, supporting B cell activation and antibody production (Brooks and Mias, 2018); Th17 cells release IL-17, driving the recruitment and activation of neutrophils, monocytes and macrophages to the site of the infection, for an efficient clearance of *S. pneumoniae* (Wilson et al., 2015; Ramos-Sevillano et al., 2019). Tregs maintain immune homeostasis by restraining excessive Th17 responses and limiting IL-17 production. An imbalance between Treg and Th17 cell populations can lead to dysregulated inflammation and has been implicated in the development of autoimmune and inflammatory diseases (Brooks and Mias, 2018). In addition to the T cells subsets mentioned above, CTLs contribute to control *S. pneumoniae* infection by directly killing infected cells (Brooks and Mias, 2018), enhancing macrophage microbicidal activity, modulating inflammatory responses, and limiting excessive tissue damage (Weber et al., 2011). Emerging evidence also highlights a contribution of CD8^+^ T cells in accelerating lung tissue repair following pneumococcal-induced injury by secreting IFN-γ, which promotes proliferation and differentiation of alveolar epithelial type II cells (Zhang et al., 2024). B cells play an essential role in both systemic and mucosal immunity during pneumococcal CAP. Following a primary infection, B cells are activated in draining lymph nodes and lung-associated lymphoid tissues, where they undergo class switching and affinity maturation. GC B cells produce high-affinity IgG and IgA antibodies that neutralize *S. pneumoniae*. Pneumococcal-specific IgA are important for controlling colonization and preventing invasion; however, *S. pneumoniae* expresses an IgA1 protease, which cleaves human IgA and impairs opsonization (Brooks and Mias, 2018). Antigen stimulation also drives the naïve B cells differentiation into IgM^+^ MBCs, and class-switched PCs that produce specific immunoglobulins needed for an efficient pathogen clearance (Brooks and Mias, 2018).

### 6.3 Immune response to *Haemophilus influenzae*

*H. influenzae* is a Gram-negative coccobacillus. Based on the presence or absence of a polysaccharide capsule, this bacterium is classified into two strains: typeable and nontypeable strains (King, 2012; King and Sharma, 2015). Most respiratory infections are caused by nontypeable strains (NTHi). In healthy adults, *H. influenzae* usually resides in the nasopharynx as part of the normal upper airway microbiome (King, 2012; King and Sharma, 2015). When it spreads to the lower respiratory tract, *H. influenzae* can become pathogenic, causing chronic obstructive pulmonary disease (COPD), bronchiectasis, cystic fibrosis, and pneumonia (King and Sharma, 2015). NTHi pneumonia is generally less severe than pneumonia caused by *S. pneumoniae* and typically occurs in individuals with underlying lung disease (King, 2012). The innate immune response to *H. influenzae* primarily involves interactions between the respiratory epithelium and recruited phagocytes. The immune surveillance begins when resident sentinel cells, mainly airway epithelial cells and alveolar macrophages, detect the pathogen. As a Gram-negative bacterium, its primary recognition is mediated by TLR4 sensing the bacterial lipooligosaccharide (LOS), often in conjunction with TLR2, which recognizes outer membrane lipoproteins (Shuto et al., 2001). When this recognition occurs, it initiates the NF-κB and p38 MAPK signaling pathways, leading to the release of antimicrobial peptides like β-defensins and the crucial neutrophil chemoattractant CXCL8. This process prompts a swift influx of neutrophils into the bronchial lumen. These neutrophils attempt to clear the infection via oxidative burst and the release of NETs, which are potently induced by NTHi presence. However, NTHi can survive within these NETs, using the DNA lattice as a scaffold to promote biofilm formation rather than being killed (Juneau et al., 2011). To persist in this inflammatory environment, *H. influenzae* has evolved multiple evasion strategies. The hallmark of NTHi persistence is its ability to form robust biofilms on mucosal surfaces, which mechanically shield the bacteria from phagocytosis and reduce the penetration of antimicrobial peptides and antibiotics (Starner et al., 2006). Beyond physical shielding, NTHi uses molecular mimicry by sialylating its LOS, thus imitating host cell structures to evade immune recognition and inhibit complement factor deposition (Hood et al., 1999). Furthermore, NTHi actively neutralizes the complement cascade by recruiting the host negative regulator factor H via its surface protein E, thereby preventing the formation of the membrane attack complex and inhibiting C3b-mediated opsonization (Singh et al., 2010). Finally, to overcome mucosal defenses, *H. influenzae* secretes specific IgA proteases that cleave secretory IgA and can invade respiratory epithelial cells via paracytosis, establishing intracellular reservoirs inaccessible to professional phagocytes and traditional clearance mechanisms (Van Eldere et al., 2014). At later stages of the infection, adaptive immunity is activated (Su et al., 2018). Once within the lung microenvironment, DCs shape T cell polarization through antigen presentation and cytokine production. In this context, Th1 and Th2 responses play complementary but distinct roles in host defense (Su et al., 2018). IL-12 produced by DCs promotes Th1 differentiation, whereas immature airway-resident DCs tend to skew T cell responses toward a Th2 phenotype. In patients with severe conditions, such as COPD or bronchiectasis, adaptive immunity shifts away from a Th1 profile (King et al., 2003). These patients exhibit diminished levels of IFN-γ and reduced CD40L expression on T helper cells, alongside increased Th2-associated cytokines. This Th1/Th2 imbalance results in suboptimal macrophage activation and impaired bacterial clearance, favoring pathogen persistence (King et al., 2003; King and Sharma, 2015). A similar shift can also be observed within the CD8^+^ T cell compartment: in COPD, Tc1 cells, which produce IFN-γ and contribute to macrophage activation and cytotoxic antibacterial responses, become functionally impaired, while Tc2 cells predominate, producing cytokines that are less effective at controlling infection. This Th2/Tc2-biased immune environment further compromises cytotoxic immunity and contributes to NTHi persistence (King and Sharma, 2015). B cells generate antigen-specific antibody responses, particularly at the mucosal level (Rao et al., 1999). Secretory IgA contributes to the bacterial neutralization and opsonization, thereby limiting the colonization of the respiratory tract and promoting pathogen clearance while shaping long-term humoral protection (Rao et al., 1999).

### 6.4 Immune response to respiratory syncytial virus

RSV is a paramyxovirus characterized by age-dependent variations in incidence and severity. Younger children are particularly susceptible, displaying higher rates of RSV-associated pneumonia and experiencing more severe clinical outcomes. In adults, RSV accounts for 4–7% of CAP cases, and older adults tend to have more severe symptoms (Pavia, 2013). Upon RSV infection, the host innate immune system initiates a defense response primarily by detecting viral genomic RNA and replication intermediates through PRRs. Beyond the cytoplasmic RIG-I pathway, the RSV surface fusion (F) protein acts as a major PAMP, stimulating TLR4 and CD14, and initiating a signaling cascade distinct from viral replication (Kurt-Jones et al., 2000). The TLR4 engagement, together with the intracellular recognition of double-stranded RNA by TLR3 and Protein Kinase R (PKR) during replication (Groskreutz et al., 2006), triggers the rapid secretion of pro-inflammatory chemokines such as IL-8 and RANTES (CCL5), orchestrating neutrophils and eosinophils influx into the airways (Harrison et al., 1999). However, RSV has evolved different evasion mechanisms. The non-structural protein NS1 targets the host ubiquitin ligase TRIM25, inhibiting the RIG-I ubiquitination and effectively silencing the RIG-I-mediated antiviral signaling (Ban et al., 2018). Furthermore, RSV proteins recruit the Elongin C-Cullin 2 complex to degrade signal transducer and activator of transcription-2 (STAT2), rendering infected cells unresponsive to type I IFNs (Elliott et al., 2007). To ensure a dampened antiviral state, RSV infection also upregulates Suppressor of Cytokine Signaling (SOCS) proteins, which inhibit the Janus kinase signaling required for interferon-stimulated gene (ISG) expression (Moore et al., 2008). Ultimately, RSV actively inhibits DCs maturation and suppresses their ability to form immunological synapses with T cells, leading to an aborted adaptive immune response (De Graaff et al., 2005). The recruitment and function of innate effector cells are actively manipulated by RSV to favor pathogenesis over clearance. Neutrophils, while abundant in RSV-infected lungs, release excessive NETs, which cause airway obstruction and lung tissue damage rather than effectively containing the virus (Cortjens et al., 2016). RSV also compromises the antiviral NK cell function through its attachment (G) glycoprotein, which contains a CX3C motif that structurally mimics the chemokine fractalkine (CX3CL1). This allows RSV to bind with high affinity to the CX3CR1 receptor on NK cells and cytotoxic T lymphocytes, thus blocking their chemotaxis and impairing their cytotoxic activity (Tripp et al., 2001). Moreover, RSV infection activates the NLRP3 inflammasome, leading to the maturation and secretion of the highly pro-inflammatory cytokine IL-1β, via ROS generation and potassium efflux (Segovia et al., 2012). Meanwhile, the pivotal orchestrators of the pulmonary immune environment, the alveolar macrophages, undergo a phenotypic shift toward an alternatively activated (M2-like) phenotype, partly driven by the IL-4Rα signaling. Although this may limit excessive tissue damage, it can inadvertently promote viral persistence and airway hyperresponsiveness rather than effective viral clearance (Shirey et al., 2010).

The adaptive immune response to RSV is critical for viral clearance and long-term immune memory but is also implicated in disease severity and immunopathology (Georgakopoulou and Pitiriga, 2025). The dynamic interplay between CD4^+^ T helper subsets (Th1, Th2, Th17, and Tregs) and CD8^+^ cytotoxic T cells influences disease outcomes by mediating the balance between effective viral clearance and the risk of excessive inflammation and lung injury (Mangodt et al., 2015). Among them, Th1 and Th2 responses exert a particularly strong influence on disease severity. A Th1 immune profile, characterized by IFN-γ production, is associated with more efficient viral clearance and milder disease, whereas a Th2-dominated response promotes airway inflammation, mucus hypersecretion, and immune dysregulation, impairing viral elimination (Mangodt et al., 2015). Consequently, an immune environment that preferentially promotes a Th2 response has been associated with more severe and clinically heterogeneous manifestations of RSV in the lower respiratory tract (Varga and Braciale, 2013; Mangodt et al., 2015; Russell et al., 2017; Gambadauro et al., 2024). Consistent with these theories, it has been demonstrated that infants with hypoxic bronchiolitis often exhibit elevated IL-4/IFN-γ ratios, indicative of Th2 skewing (Varga and Braciale, 2013; Mangodt et al., 2015; Russell et al., 2017; Gambadauro et al., 2024). Furthermore, elevated IL-17 levels detected in both tracheal aspirates and plasma of children with severe RSV infection, indicate an active involvement of Th17-associated pathways in disease (Mangodt et al., 2015). In this regard, a comparative analysis of non-ventilated versus ventilated infants infected with RSV showed elevated plasma levels of IL-17 in non-ventilated subjects. This finding indicates that children experiencing more severe clinical outcomes may exhibit an exhausted inflammatory response (Larrañaga et al., 2009; Faber et al., 2012). IL-17 contributes to RSV immunopathology by promoting mucus overproduction and airway obstruction, amplifying Th2 cytokine activity, and driving neutrophil recruitment and accumulation in the lung (Varga and Braciale, 2013). Moreover, IL-17 negatively regulates the transcription factors T-bet and Eomes, which are essential for CD8^+^ T-cell differentiation and effector functions. This suppression compromises antiviral cytotoxic responses causing delayed viral clearance and tissue damage (Mukherjee et al., 2011; Mangodt et al., 2015).

During acute RSV infection, Treg numbers in the lung increase significantly (Varga and Braciale, 2013; Christiaansen et al., 2014). Experimental depletion of Tregs exacerbates pulmonary inflammation, characterized by elevated IL-6 production and enhanced recruitment of macrophages and neutrophils into the airways (Ruckwardt et al., 2009; Fulton et al., 2010; Lee et al., 2010).

Regarding B cells, primary antibody response to RSV infection initiates in the lymph nodes draining the respiratory tract, where Tfh cells provide signals for B cell activation (Varga and Braciale, 2013; Gambadauro et al., 2024). As a result, B cells proliferate and differentiate into PCs that secrete RSV-specific antibodies, as well as MBCs primed for future encounters. Both mucosal IgA and serum IgG play a neutralizing role by targeting RSV attachment (G) and fusion (F) glycoproteins (Varga and Braciale, 2013). However, RSV has evolved several strategies to evade B-cell-mediated humoral immunity. For instance, the RSV G glycoprotein displays structural diversity and extensive glycosylation, which together can mask key antigenic sites. It also acts as an immunological decoy by sequestering neutralizing antibodies and redirecting them away from conserved viral epitopes, thereby impairing effective antibody-mediated neutralization (Bukreyev et al., 2008). Moreover, RSV infection is associated with delayed or suboptimal antibody affinity maturation and impaired MBCs formation, contributing to the high susceptibility to reinfection (Georgakopoulou and Pitiriga, 2025).

### 6.5 Immune response to SARS-CoV-2

SARS-CoV-2 is the coronavirus responsible for COVID-19. SARS-CoV-2 pneumonia is characterized by extensive lung inflammation that can progress to respiratory distress and lead to several correlated complications. Although most individuals experience only mild symptoms, before the massive vaccination and the onset of the Omicron variant, approximately 14% of infected individuals developed severe symptoms requiring hospitalization due to pneumonia (Adil et al., 2021; Caliman-Sturdza et al., 2025). Among these, 5–10% progressed to a critical state necessitating intensive care support, often including mechanical ventilation due to acute respiratory distress syndrome (ARDS) (Caliman-Sturdza et al., 2025).

Upon entering the host, SARS-CoV-2 is detected by innate immune sensors such as TLR7 and melanoma differentiation-associated protein 5 (MDA5), which recognize viral RNA and trigger antiviral defenses. However, a hallmark of severe COVID-19 is a unique immune dysregulation characterized by a profoundly suppressed type I IFN response juxtaposed with excessive production of pro-inflammatory cytokines (Blanco-Melo et al., 2020; Aiello et al., 2021; Petrone et al., 2021; Petruccioli et al., 2021; Aiello et al., 2022). RNA-seq analyses have demonstrated that patients with mild COVID-19 present a robust type I IFN response and reduced levels of type III IFNs in both upper and lower respiratory tract, whereas patients with a severe disease form exhibit a defective IFN signaling and a reduced induction of ISGs (Bastard et al., 2022). In some individuals, this impairment is linked to the presence of neutralizing autoantibodies against type I IFNs at mucosal sites, underscoring the critical role of early IFN-mediated antiviral immunity in controlling SARS-CoV-2 replication in the upper airways (Bastard et al., 2022). This delay in antiviral signaling is orchestrated by multiple viral proteins that disarm host defenses. Notably, the non-structural protein 1 (Nsp1) binds to the host 40S ribosomal subunit and shuts down the translation of host mRNAs, including those encoding interferons, effectively silencing the cell’s alarm system at the level of protein synthesis (Thoms et al., 2020). Concurrently, the viral protein ORF6 blocks the nuclear transport of STAT1 and STAT2, the two transcription factors essential for interferon signaling, rendering cells refractory to any external antiviral warning signals (Mu et al., 2020). In the absence of effective viral control, the immune system compensates with a pathological hyper-inflammatory reaction driven by the NLRP3 inflammasome. SARS-CoV-2 activates this cytosolic complex, which is strongly associated with disease severity and lung injury, by inducing the release of high levels of IL-1β and IL-18 (Rodrigues et al., 2021). This inflammatory cascade further recruits neutrophils that release NETs in a dysregulated manner, contributing to the immune-thrombosis and microvascular occlusion often observed in severe cases (Zuo et al., 2020). Similar to RSV, SARS-CoV-2 manipulates the myeloid compartment, causing a depletion of non-classical monocytes and an accumulation of dysplastic, HLA-DR^low^ monocytes that perpetuate cytokine storm while failing to present antigens effectively (Schulte-Schrepping et al., 2020). Patients with severe COVID-19 exhibit a marked functional exhaustion of NK cells and CTLs, which not only decrease in number but also upregulate the inhibitory receptor NKG2A, hindering their ability to produce CD107a, IFN-γ and IL-2, and thereby preventing the clearance of infected cells (Zheng et al., 2020). Simultaneously, the innate response is also amplified by pathological activation of the complement system, specifically through the C5a-C5aR1 axis; the binding of the anaphylatoxin C5a to its receptor C5aR1 on myeloid cells drives a potent pro-inflammatory feedback loop leading to acute lung injury and thrombotic complications (Carvelli et al., 2020). Moreover, single-cell RNA sequencing of bronchoalveolar lavage fluids reveals a critical shift in the lung microenvironment with the progressive depletion of the beneficial, tissue-resident alveolar macrophages (FABP4-positive) and their replacement by highly inflammatory, monocyte-derived macrophages (FCN1-positive), which propagate the cytokine storm rather than resolve the infection (Liao et al., 2020). Regarding the adaptive immune response, SARS-CoV-2 infection engages all major components, including antibody-producing B cells, helper CD4^+^ T cells, and CTLs. Collectively, these responses are essential for shaping infection trajectory, viral control, and clinical recovery (Aiello et al., 2023). Importantly, the timing of adaptive immune activation is a critical determinant of COVID-19 outcome. Early induction of robust CD4^+^ and CD8^+^ T-cell responses is consistently associated with mild disease, rapid viral clearance, and protection from excessive inflammatory damage. Individuals who mount rapid T-cell responses, marked by effective CD4^+^ T-cell help and potent CD8^+^ cytotoxic activity, limit viral replication in the respiratory tract. In contrast, delayed T cell responses allow uncontrolled viral proliferation, increasing the probability of severe disease and systemic inflammation (Sette and Crotty, 2021; Silva et al., 2022; Iwanaga et al., 2023; Petrone et al., 2023; Sette et al., 2023). Under these circumstances, adaptive responses develop in a host milieu marked by high viral loads and extensive inflammation, conditions that collectively contribute to reducing immune effectiveness (Sette and Crotty, 2021; Iwanaga et al., 2023). As a result, Th1, Th2, Th17, and Treg cells are generated but show deficient effector functions (Silva et al., 2022). In SARS-CoV-2 infection, CD4^+^ T cells commonly adopt a Th1 or Tfh profile. Through the production of IL-21, a canonical Tfh-derived cytokine, CD4^+^ T cells also aid CD8^+^ T-cell responses (Sette and Crotty, 2021). In the context of B cell responses, Tfh cells are essential orchestrators of the GC reaction and are indispensable for the establishment of a robust humoral immune memory. However, GC response requires approximately two weeks to develop, a long time in the context of a viral infection. Longitudinal studies tracking B cell responses show that the MBCs compartment expands and remains relatively stable for 6–12 months after infection (Piano Mortari et al., 2025). Following natural infection, in addition to T cells, memory B cells persist for months providing durable protection against severe disease (Piano Mortari et al., 2025). In severe COVID-19, dysregulated EF response, including expansion of the double-negative (DNs) B cells and short-lived PCs, have been linked to hyper-inflammation and tissue damage (Woodruff et al., 2020; Lee et al., 2022). This extrafollicular activation is associated with a rapid expansion of antibody-secreting cells and the early production of high concentrations of SARS-CoV-2–specific neutralizing antibodies (Turner et al., 2021).

### 6.6 Immune response to influenza A virus and influenza B virus

Influenza viruses belong to the family *Orthomyxoviridae* and are characterized by a segmented, negative-sense, single-stranded RNA (ssRNA) genome. Their classification is based on antigenic and genetic differences, which divide them into four genera: influenza A, B, C, and D (Chen et al., 2018). IAV is further subdivided according to the molecular structure and genetic properties of its two surface glycoproteins, hemagglutinin (HA) and neuraminidase (NA) (Chen et al., 2018). The subtypes most associated with human infection are H1N1, H1N2, and H3N2. Infection with IAV generally manifests as a mild respiratory illness, predominantly affecting the upper respiratory tract. However, it can progress to lower respiratory tract infections that pose a high risk of severe disease in older adults (Gambadauro et al., 2024). Despite receiving less scientific attention than IAV, IBV remains a significant contributor to global seasonal influenza burden (Hensen et al., 2020). IBV strains are classified into two distinct lineages based on antigenic and phylogenetic characteristics: B/Victoria/2/87 and B/Yamagata/16/88 (Caini et al., 2019). The innate immune system rapidly detects IAV primarily through the cytosolic sensor RIG-I, which recognizes the 5’-triphosphate moiety present on viral genomic ssRNA and triggers a signaling cascade that culminates in the type I IFN production (Pichlmair et al., 2006). However, the virus possesses a potent antagonist in its non-structural protein 1 (NS1), which directly interacts with the host ubiquitin ligase TRIM25. By inhibiting RIG-I ubiquitination, NS1 prevents the conformational changes required for downstream signaling, thereby interrupting the antiviral response at its onset (Gack et al., 2009). Furthermore, NS1 broadly suppresses host gene expression by binding the 30-kDa subunit of the cleavage and polyadenylation specificity factor (CPSF30), which blocks the processing and nuclear export of host mRNAs, including those encoding interferons and antiviral proteins (Nemeroff et al., 1998). Beyond immune evasion, IAV infection engages the NLRP3 inflammasome through the recognition of viral RNA, leading to the maturation of IL-1β and IL-18. While this contributes to viral clearance, excessive inflammasome activation is often associated with severe lung pathology (Allen et al., 2009). In highly pathogenic strains, such as the 1918 H1N1 or H5N1 viruses, the viral protein PB1-F2 further exacerbates the inflammatory landscape by targeting mitochondria and increasing susceptibility to secondary bacterial infections, contributing to a dysregulated cytokine storm (McAuley et al., 2007). Ultimately, this excessive cytokine amplification is often orchestrated not only by immune cells, but also by pulmonary endothelial cells, which become the major producers of the inflammatory milieu that causes vascular leakage and acute respiratory distress (Teijaro et al., 2011). In addition to the initial cytokine response, innate immune effectors cells play a dual role in both protection and pathogenesis. NK cells are critical for early viral containment, directly recognizing infected cells through the interaction between the viral surface HA and the natural cytotoxicity receptor NKp46, a mechanism that triggers immediate lysis of infected cells (Mandelboim et al., 2001). However, this protective response is often overwhelmed by a massive and dysregulated influx of neutrophils into the alveolar space. In fatal cases of influenza, these neutrophils release excessive NETs which become physically entangled with the alveolar epithelium, causing extensive tissue injury and obstructing gas exchange (Narasaraju et al., 2011). Another feature of severe influenza is the rapid necrosis and subsequent depletion of resident alveolar macrophages. The loss of the lung’s primary phagocytic defense not only impairs viral clearance but also creates an “immunological empty niche” that makes the host highly susceptible to life-threatening secondary bacterial superinfections, such as those caused by *S. pneumoniae* (Ghoneim et al., 2013).

Regarding the adaptive immune response to IAV, CTLs play a central role in clearing viral infection and aiding recovery by producing antiviral cytokines and directly killing IAV-infected cells (Gambadauro et al., 2024). CTLs recognize short peptide fragments derived from conserved viral proteins presented by MHC class-I molecules, providing broad cross-reactive immunity (Gambadauro et al., 2024). Upon activation, CTLs initiate an effector program that limits viral spread by firstly releasing cytotoxic granules containing perforin and granzymes, which induce apoptosis in infected cells (Chen et al., 2018). Perforin acts by forming pores in the target cell membrane, facilitating the entry of granzymes into the cytosol and thereby promoting apoptosis (Chen et al., 2018). Among these, granzyme A (GrA) contributes to antiviral defense by inhibiting viral replication through the cleavage of viral and host proteins (Chen et al., 2018). Secondly, CTLs can induce apoptosis through death receptor–mediated pathways by expressing ligands such as Fas ligand (FasL/CD95L) and TRAIL, which engage their corresponding receptors on infected cells. Thirdly, CTLs secrete pro-inflammatory cytokines, including IFN-γ and TNF-α, which amplify and coordinate the immune response enhancing antiviral activity (Braciale et al., 2012; Koh et al., 2023). Notably, at the peak of IAV infection, CD8^+^ T cells also produce immune-regulatory cytokines such as IL-10, which help limit excessive inflammation and lung tissue damage, thereby balancing effective viral clearance with host protection. After an initial encounter with IAV, CTLs circulate through the blood, lymphoid tissues, and the site of infection, remaining ready to mount a rapid response upon re-exposure (Carvelli et al., 2020). In parallel, CD4^+^ T cells subsets, such as Th1 and Tfh cells, help fight IAV infection. Th1 cells secrete IFN-γ and IL-2 to enhance CTL and macrophage responses, while Tfh cells support B cell activation (Chen et al., 2018; Gambadauro et al., 2024). Once activated, B cells undergo clonal expansion and differentiate into PCs that secrete IAV-specific antibodies, primarily targeting surface glycoproteins HA and NA. While IgA provide important protection at mucosal surfaces of the respiratory tract, IgG represent the main antibody class mediating systemic defense against influenza. Evidence suggests that IgG help limit disease severity, whereas IgA are particularly effective at reducing viral transmission (Gambadauro et al., 2024). Additionally, IAV has developed various strategies to avoid host immune responses, including rapid mutations in HA and NA that allow the virus to escape recognition by pre-existing antibodies and avoid neutralization. Through antigenic drift and antigenic shift, IAV continually generates new variants with altered antigenic features, complicating its immune recognition and contributing to recurrent seasonal epidemics and occasional pandemics (Gambadauro et al., 2024). Regarding IBV, it is initially detected by the host through the same RIG-I-dependent recognition of viral RNA used by IAV. The virus has evolved different strategies to subvert this defense, largely mediated by its unique NS1. Unlike IAV NS1 that primarily targets TRIM25, IBV NS1 specifically binds to the host ubiquitin-like molecule ISG15 (Interferon-Stimulated Gene 15), preventing the ISGylation of host proteins, a key post-translational modification required to signal the presence of the pathogen and amplify the antiviral state (Yuan, 2001). Furthermore, IBV NS1 blocks the host nuclear transport machinery to suppress immune gene expression by binding the nucleoporin Nup98, thereby inhibiting the nuclear export of host mRNAs and preventing the translation of antiviral cytokines (Schneider and Wolff, 2009). Beyond these post-transcriptional interferences, IBV actively intercepts the signaling pathway immediately downstream of PRR by directly interacting with the interferon regulatory factor (IRF) 3, effectively preventing its nuclear translocation and the subsequent induction of the IFN-β promoter (Donelan et al., 2004). Furthermore, the innate defense against IBV relies on NK cells. In this regard, the IBV HA is directly recognized by the natural cytotoxicity receptor NKp46, triggering the lysis of infected cells regardless of prior antibody sensitization (Mandelboim et al., 2001). A distinct feature of the innate response to IBV involves the kinetic of the macrophage response. Unlike seasonal IAV strains, which often delay immune recognition, IBV infection of human macrophages induces a significantly more rapid and robust pro-inflammatory cytokine response, particularly involving IL-6 and TNF-α, which likely contribute to the clinical severity often observed in pediatric patients (Österlund et al., 2012). While the roles of CD8^+^ and CD4^+^ T cells in protection against IAV are well established in both animal models and humans, much less is known about the contribution of T cell–mediated immunity to IBV infection.

## 7 Clinical addendum: immunomodulation to optimize the innate-adaptive transition in community-acquired pneumonia

In severe CAP, the strongest clinical evidence for improving outcomes through immunomodulation involves controlling early innate hyper-inflammation. This strategy is more effective than attempts to accelerate adaptive immune responses. Randomized trials indicate that, in carefully selected patients with severe CAP, particularly those exhibiting high levels of inflammation, adjunctive corticosteroids reduce treatment failure and may decrease short-term mortality (Dequin et al., 2023). These findings support the mitigation of tissue-damaging innate responses to preserve lung function during the initiation of pathogen-directed therapy. Conversely, interventions aimed at enhancing innate effector functions, such as inhaled or systemic granulocyte-macrophage colony-stimulating factor (GM-CSF) or inhaled IFN-β, remain mechanistically promising but lack clinical validation in bacterial CAP (Mathias et al., 2015). These approaches have produced inconsistent results in viral pneumonias and acute respiratory distress syndrome (ARDS). Current evidence favours a precision-based immunomodulation approach in CAP by identifying patients with severe disease and pronounced inflammation, as short-course corticosteroids can improve outcomes in this subgroup (Dequin et al., 2023).

## 8 Challenges and future directions

Despite substantial advances in antimicrobial therapy and supportive care, CAP remains a significant public health challenge, largely due to the increasing prevalence of multidrug-resistant pathogens and the aging of the global population (Vaughn et al., 2024). While the recent integration of multiplex PCR panels and NGS has drastically improved our ability to detect respiratory pathogens, significantly reducing non-detection rates in complex settings, a critical unmet need remains: translating these molecular data into effective antimicrobial stewardship (Liu et al., 2024). The mere identification of a pathogen is often insufficient; future diagnostic strategies must increasingly focus on the rapid and simultaneous detection of resistance markers to guide precise empirical treatment and curb the spread of antimicrobial resistance (McCullers, 2006). A major emerging area of investigation concerns the complex biological synergy in polymicrobial infections. As highlighted, respiratory viruses often act as “pathogen-facilitators” by depleting alveolar macrophages and disrupting epithelial barriers, thereby creating an “immunological empty niche” that predisposes hosts to severe secondary bacterial superinfections (McCullers, 2006). Moving forward, research must shift from studying single-pathogen models to elucidating the cross-talk between viral and bacterial agents. Understanding how initial viral insults alter the lung microenvironment to favor bacterial persistence could reveal novel therapeutic targets aimed at blocking the transition from viral infection to severe bacterial pneumonia, rather than relying solely on antibiotics once superinfection is established (Holter et al., 2015). Current evidence suggests that in severe CAP, mortality is often driven by a dysregulated host response characterized by hyper-inflammation and cytokine storms, rather than solely by the pathogen burden, as described in COVID-19. While broad immune-stimulatory approaches have shown inconsistent results, selectively targeting specific inflammatory pathways offers a more refined strategy (Mathias et al., 2015; Cantini et al., 2020; Goletti and Cantini, 2021; Ferraccioli et al., 2022; Alonzi et al., 2024). The integration of immune biomarkers, such as sTREM-1 or pro-adrenomedullin, into clinical practice could allow the identification of specific patient phenotypes most likely to benefit from immunomodulatory therapies (Dequin et al., 2023). Ultimately, the future of CAP management will likely depend on “theragnostic” approaches that simultaneously target the invading pathogen and tailor the host immune response to prevent tissue damage while ensuring effective clearance (Gibot et al., 2004; Liu et al., 2016).
